# Attitudes of medical students regarding legalisation of cannabis and cannabis-education

**DOI:** 10.4102/sajpsychiatry.v29i0.1948

**Published:** 2023-11-07

**Authors:** Evan Eiselen, Kalaivani Naidu, Maryn Viljoen

**Affiliations:** 1 Department of Psychiatry, Faculty of Medicine, University of Pretoria, Pretoria, South Africa; 2 Private Statistician, Luckhoff, South Africa

**Keywords:** legalisation, cannabis, medical student, medicinal cannabis, recreational cannabis, medical training curricula

## Abstract

**Background:**

Recreational and medicinal use of cannabis is topical in the light of more permissive legislation regarding the substance worldwide.

**Aim:**

The primary purpose of this study is to determine the attitudes that final-year medical students at the University of Pretoria (UP) hold about recreational and medicinal use of cannabis, as well as determining if they feel they are being adequately trained in this regard.

**Setting:**

The research was conducted at Weskoppies Psychiatric Hospital, affiliated with the UP.

**Methods:**

The study follows a cross-sectional, comparative, quantitative design. Data were collected by means of a structured questionnaire. Final-year medical students were identified as participants via a convenience sampling technique. Participation was voluntary and anonymous.

**Results:**

A total of 57 valid responses were recorded. The study shows that most medical students had permissive views about cannabis and that the majority feel that they are not being adequately trained to advise patients about medical cannabis in a lecture setting (64.9%, *n* = 37) or clinical setting (68.4%, *n* = 38). Results also show that previous personal experience with cannabis led to more permissive views.

**Conclusion:**

This study illustrates the need for academic research regarding medicinal cannabis but interestingly shows that medical students want more guidance from their training institution about the topic.

**Contribution:**

This research shows that the conversation surrounding cannabis in medicine is continuous and universities should make a conscious effort to familiarise students with the topic.

## Introduction

Over the past few years, South Africa has experienced a major shift in terms of cannabis laws. Following the ruling of the Constitutional Act in September of 2018, Sections 4(b) and 5(b) of the *Drugs and Drug Trafficking Act* as well as Section 22A(9)(a)(i) of the *Medicines and Related Substances Act,* it was found that an individual’s fundamental right to privacy was encroached upon and therefore deemed unconstitutional.^1^

The global trend towards cannabis use seems to be that people are more interested in its use, both recreational and medicinal, and less critical or conservative in their view of the previously often vilified drug.^2,3,4^ This means that practitioners will increasingly be confronted with patients seeking information about the validity and safety of using cannabis for different ailments in the near future. This poses two questions: firstly, what is a practitioner’s personal view on using cannabis as well as what factors form this particular view? and secondly, does the practitioner feel like they have an adequate knowledge base about cannabis to confidently advise their patients?

It is also important to note that relaxing the cannabis laws in a country or state may lead to more permissive views among its citizens.^4^ A recent study done in the United States, focusing on the attitudes of young adults, found that the youths living in states where cannabis has been legalised (for either medicinal or recreational use) had significantly more liberal ideas about the drug than their peers living in states where cannabis is still illegal.^5^ It is also suggested that attitudes and beliefs about substances are forged during the late adolescence and early adulthood period of life.^6,7^ The study also illustrated the general trend towards a more open-minded approach concerning cannabis among the American youth, finding that young adults in 2013 are almost two-and-a-half times more likely to have permissive views about the drug when compared to the same age group in 2004.^5^ Again it seems important then to have an idea about what our own future practitioners, as young adults, think when it comes to cannabis.

There are a significant number of studies that examine the viewpoint that already established and practising medical doctors hold about medical cannabis, but few that look into the beliefs of future practitioners. A literature search found only two studies that were conducted by Chan et al.^3^ in America and Vujicic et al.^2^ in Serbia that specifically focus on medical students’ attitudes towards the drug and both had similar results. Both of these studies were done using questionnaires that enquired about a few main themes, including previous use of cannabis, current knowledge about cannabis (both clinical indications for its use and side effects) and lastly their personal view with regard to both the medicinal and recreational use of marijuana. These two studies were conducted at the University of Colorado and the University of Belgrade, respectively.^2,3^

Results from these studies showed that the majority of students were in favour of the legalisation and medicinal use of cannabis that male students are significantly more likely to try the drug than their female peers and that students who had previously experimented with cannabis held more liberal views about the drug’s legalisation and use as opposed to students who have not. Furthermore, the studies also showed that students who had experimented with cannabis displayed more knowledge about the conditions that cannabis could be used for, conversely students who had no experience with the drug had more knowledge pertaining to cannabis’s side effect profile.^2,3^

Furthermore, Vujicic et al.^2^ asked the students to identify sources from which they attained knowledge about medical marijuana. Internet and television were found to be the places where students found most of their information, with formal university education falling far behind. Accordingly, it can be argued that tertiary institutions need to assess whether they are adequately preparing and equipping future doctors with the necessary knowledge and skills when it comes to the controversial topic of medical marijuana.

A recent study conducted at the University of the Free State looked at the prevalence of cannabis and tested knowledge pertaining to the negative side effects of cannabis in the undergraduate medical student group. Results showed that although lifetime prevalence and patterns of cannabis use are similar to those reported by other South African studies (not specific to medical students), medical students consistently had poor knowledge about the negative side effects associated with the drug.^8^ When we look at research conducted elsewhere in Africa, we see a different reality. A study conducted in Nigeria showed that the majority (84.5%) of fifth-year medical students who participated did not agree with legalisation of cannabis and the majority had conservative views about cannabis use as well as the use of other psychoactive substances.^9^

The primary objective of this study was to determine the attitudes of final-year medical students enrolled at the University of Pretoria (UP) towards the legalisation of cannabis. Secondary objectives included determining the attitudes and knowledge that these same students have with regard to the medicinal and recreational use of cannabis, investigating the effects of previous cannabis use on the students’ attitudes and knowledge about cannabis. The authors also wanted to determine whether students at the UP are of the opinion that the current curriculum adequately prepares them when it comes to the issue of medical cannabis. Lastly, the results of this study will also be compared to the results of studies done abroad.

## Research methods and design

### Study design

This study was a cross-sectional and quantitative study. Data were collected from participants using a specifically designed questionnaire that contained questions about demographics, personal experience with cannabis and views and/or attitudes about legalisation of cannabis as well as the medical and recreational use of cannabis. This questionnaire was self-designed keeping the aims of the study in mind and consists of 34 questions. The questionnaire was not based on a standardised scale and was rather assessed subjectively through face validity.

### Setting

This research was conducted at Weskoppies Psychiatric Hospital. Weskoppies Hospital (WKH) is a tertiary specialist psychiatric hospital located in Pretoria West, Gauteng and is affiliated to the UP as well as the Sefako Makgatho Health Sciences University and Nursing colleges.

### Study population and sampling strategy

Student intern complex (SIC) students are medical students, also called student interns, who are completing the last 18 months of their training at the university before being employed as Intern Doctors in the Department of Health. Student intern complex groups consist of both fifth-year and final-year medical students, meaning that approximately half the group would already have completed a clinical rotation in psychiatry and the other half’s first exposure to such a rotation. This particular group of students made up the study population of the research project. In light of the coronavirus disease 2019 (COVID-19) pandemic, large gatherings were not possible for data collection and online questionnaires have markedly low response rates.

The original plan was to gain maximal participation by distributing the questionnaire to the entire final-year student group to complete after they had their block orientation. However, the pandemic curtailed this plan and forced the researchers to change the methodology of the study. Accordingly, a convenience sampling technique was used to select students for the study, ultimately weakening the validity of the date because of a smaller sample size, but allowing research to continue in a safe way, abiding by the rules set in place by the University.

Inclusion criteria for the participants included that they have to be a registered SIC student at the UP as well as willing to participate voluntarily and able to provide informed consent at the time of completing the questionnaire. Exclusion criteria included not being a registered SIC student or being unwilling to participate or provide informed consent.

These data were collected between two SIC groups that rotated through WKH between January 2021 and March 2021 in a lecture room setting, meaning that the participants completed the questionnaire immediately as they received it. There was a total of 84 possible participants (42 in each group), a total of 61 students participated, but only 57 responses were valid. Four questionnaires were either incomplete or participants gave more than one answer in questions where only one response was asked.

### Data collection

The instrument for data collection was a specifically designed, self-compiled questionnaire and contained questions divided into the following headings: (1) demographics, (2) attitude towards legalisation of cannabis, (3) knowledge and opinion on use of cannabis both recreational and medicinal, (4) adequate training and/or education regarding cannabis and (5) personal use and/or experience with cannabis. Please see the included tables for a more comprehensive view of the questions asked.

Each questionnaire also had an informed consent form that had to be completed for the questionnaire to be valid. The questionnaires were distributed in person by the primary investigator to participating students, keeping all social distancing and other precautions in mind. The participants were able to raise any doubts or concerns about the questions to the primary investigator for clarification.

### Data analysis

Data from the questionnaires were captured electronically by the researcher and any further analysis was done by a statistician using SAS version 9.2. Descriptive statistics, namely frequencies and percentages, were calculated for categorical data, and means and standard deviations or medians and percentiles were calculated for continuous data. The Shapiro-Wilk test was performed to investigate whether continuous data such as age followed a normal distribution.^10^ In this study, Cronbach’s alpha (α) was used to calculate the internal consistency of items in the subscales (attitude towards legalisation of cannabis, knowledge and opinion on the use of cannabis both recreational and medicinal as well as personal use and/or experience with cannabis) in the questionnaire. According to George and Mallery, a Cronbach’s alpha value above 0.90 indicates excellent internal consistency, above 0.80 is good, above 0.70 is acceptable, above 0.60 is questionable, above 0.50 is poor and below 0.50 is unacceptable.^11^ Analytical statistics namely Fisher’s exact test was used to compare proportions between groups. A significance level (ɑ) of 0.05 was used in this study, meaning a level of less than 0.05 would be significant.

### Ethical consideration

The study was approved by the Faculty of Health Sciences Research Ethics Committee of the University of Pretoria (reference number: 621/2020). The Executive Committee of the School of Medicine gave permission to include students in the study. Written informed consent was obtained from each participant prior to distribution of the questionnaire and participants were informed that they could withdraw from the study at any point.

## Results

As mentioned previously, there were a total of 61 responses out of a possible 84, resulting in a participation rate of 72.6%. Fifty-seven of these responses were valid constituting a response rate of 93.4%. The study sample consisted of 21 male (36.84%) and 36 female (63.16%) students. According to the Shapiro-Wilk test for normality, the distribution of the age of the participants did not follow a normal distribution (*W* = 0.800, *p* < 0.0001). Consequently, the median (24 years) and the interquartile range (lower quartile = 23 years, upper quartile = 26 years) are reported on. Regarding other qualifications, the majority of students (82.46%) had no prior qualifications, eight students (14.04%) had obtained a Bachelor’s degree and only two students (3.5%) had either a Master’s or an Honours degree.

**FIGURE 1:**
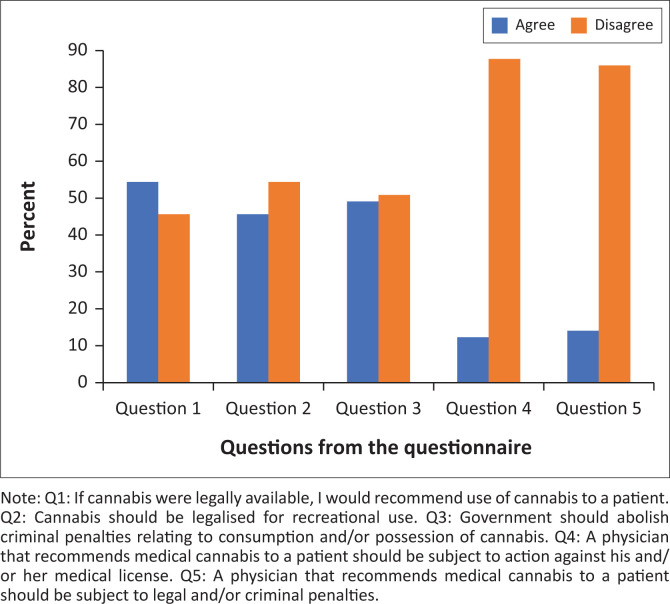
Attitudes about legalisation of cannabis.

The first subscale of the questionnaire included questions about the participant’s attitudes towards the legalisation of cannabis and the reliability analysis resulted in a Cronbach’s alpha of 0.65, indicating questionable internal consistency. Figure 1 presents a summary of the results.

**FIGURE 2:**
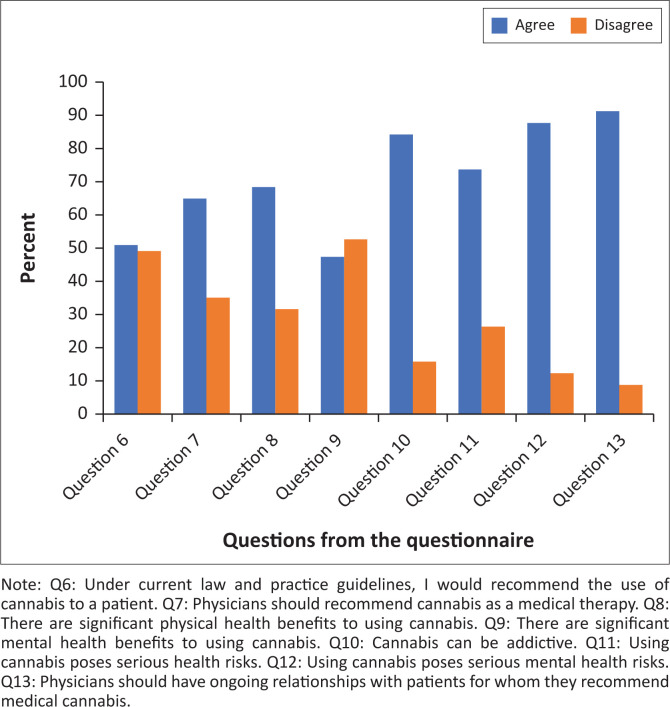
Knowledge and opinions on recreational and/or medicinal use of cannabis.

The next subscale comprised questions about the participant’s knowledge and opinion on the use of cannabis, both recreationally and medicinally. The Cronbach’s alpha for this subscale was 0.7, indicating acceptable internal consistency. A breakdown of the results of this subscale is represented in Figure 2.

Of the participants, just over half (*n* = 29, 50.9%) mentioned that they would recommend cannabis to a patient under current law and practice guidelines. It also showed that 68.4% (*n* = 39) of participants believe that cannabis has significant physical benefits but 42.9% (*n* = 30) believe that cannabis does not have any significant mental health benefits.

**FIGURE 3:**
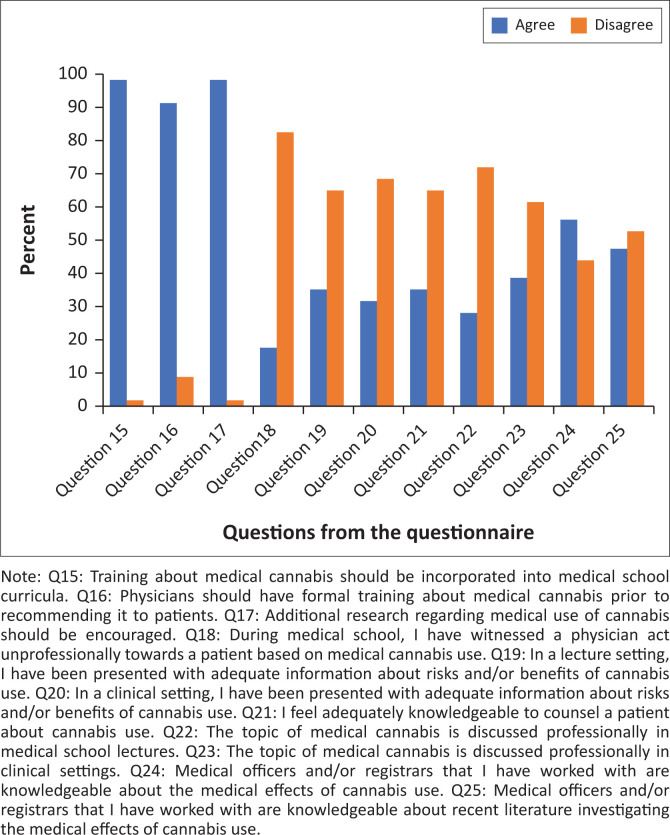
Opinion about adequate training and/or education regarding cannabis.

The third section of the questionnaire investigated the participant’s opinion about adequate training and education regarding cannabis. The majority of students (*n* = 56, 98.2%) agreed that teaching about cannabis should be included in the medical school curricula and the entire study population agreed that physicians should receive formal training prior to recommending cannabis to patients. Detailed answers to this subscale are represented in Figure 3.

When asked what sources students used to gain information about cannabis, they indicated that 31.6% (*n* = 18) gained information mainly from television, radio and media articles, 21.1% (*n* = 12) indicated information was gained from the recreational use of cannabis by their friends and/or family and 19.3% (*n* = 11) said that medical literature was their main source of information. Classroom lectures and information from senior colleagues (medical officers and/or registrars and/or consultants) were only shown to be six students’ (10.5%) main source of information about cannabis.

The last section of the questionnaire investigated students’ personal use and/or experience with cannabis. Of the 57 participants, 33 (57.9%) indicated that they have used cannabis in the past. Of those 33 students, 18 had used cannabis in the past year, 7 had used cannabis in the past month and 4 had used cannabis in the past week, leaving 4 students who used cannabis more than a year ago. The majority of these students indicated that they used cannabis for recreational purposes (*n* = 29, 87.9%) and the remaining 12.1% (*n* = 4) reports using it for both medicinal and recreational purposes.

Students who had any personal experience with cannabis were compared to those who had never used the drug before and how that might influence their attitudes and views about the legalisation of cannabis. It was found that students who had prior personal experience with cannabis were more in favour of legalising cannabis, recommending cannabis to patients and were against practitioners being subject to legal or criminal penalties for recommending cannabis to patients. There was, however, no significant difference between the two groups of students (*p* = 0.3955).

Significant differences were seen with an increase in the frequency of use. Students that had used cannabis in the past month compared to students with no personal experience with cannabis had significant differences with regard to their views towards legalisation of cannabis. These differences were even more pronounced between students that had used cannabis in the past week and those who had not used cannabis at all.

## Discussion

As mentioned earlier, globally there is an increase in more permissive views about cannabis as well as rapidly changing policies regarding the possession and use thereof. An increasing amount of countries around the world have legalised cannabis use for specific groups of patients, or in some instances complete decriminalisation that allows for recreational use of cannabis. Germany, Italy, Spain, France, Romania, the Czech Republic, Colombia and 23 states in the United States have already legalised some form of medical cannabis.^12^ Other countries that have followed suit include: Canada, Austria, the Netherlands, Portugal, Finland and Israel.^13^ Certain studies also illustrate a relationship between decriminalisation policies and more permissive views about cannabis, especially in adolescents and young adults.^4,5^ This is an important point taking into account that the attitudes and beliefs people foster about substances are formed during late adolescence and early adulthood. It is also important to note that young adults are likely to be the most affected when it comes to more permissive policies regarding cannabis seeing that they are of voting age and thus more attuned to policy debates. They are also at the peak years of possible exposure and engagement with cannabis across their lifespan.^14,15^

There is not a vast amount of literature investigating cannabis use in medical students, but a systematic review and meta-analysis conducted in 2018 and consisting of 38 studies showed a lifetime prevalence of cannabis use of 31.4% among medical students, past-year use of 17.2% and past-month use of 8.8%.^16^ These results are similar to the results obtained in two studies, conducted in Serbia and Colorado (USA) about attitudes of medical students surrounding the legalisation of cannabis.^2,3^ Similar to these two studies, the majority of participants in this study had favourable views about the medicinal use of cannabis and felt that medical practitioners should not be at risk of criminal prosecution if they recommend cannabis to a patient.

Cannabis education was the second theme to be explored in this study, focusing on what sources students used to obtain information about cannabis and whether they felt like they were being adequately prepared to counsel and advise patients regarding its use. The majority of students (*n* = 56, 98.2%) agreed that medical schools should include cannabis education in their curricula, yet a large proportion (*n* = 37, 64.9%) expressed that they felt they have not received adequate training in a lecture setting. A further 38 students (68.4%) expressed the same sentiment about cannabis education in a clinical setting. This dilemma may be exacerbated by the fact that just under half of the participants felt that the medical officers and registrars working with them were not knowledgeable enough about the medical effects of cannabis use and were not up to date with the most recent literature regarding medicinal cannabis.

It was also important to evaluate what sources students use in order to gain information about cannabis. This study found that the majority, accounting for just over a third, used mainstream media (television, radio and media articles) as their main source of information, which is concerning when comparing it to medical literature (19.3%, *n* = 11) and classroom lectures and/or information from senior colleagues (*n* = 6, 10.5%). This may suggest that there are too few medical education opportunities with regard to cannabis. It is therefore unsurprising that the majority (*n* = 37, 64.9%) felt like they were not knowledgeable enough to counsel a patient about cannabis use. These findings are consistent with international studies conducted about the same topic^2,3^ and require further investigation, but one can safely assume that tertiary institutions should focus on including more cannabis-related education in their curricula. All students agreed that further research regarding cannabis and its effects needs to be conducted.

One must also consider the amount of training that students receive in the current curriculum regarding cannabis and its effects. Currently students rotate through a psychiatry block in fifth year that covers general psychiatry including substance-related disorders, followed by a clinical rotation in their final year lasting another 8 weeks. Students are provided with an ample amount of information regarding cannabis in the psychiatry sphere, but it is not known from the researchers’ perspective how well cannabis education extends throughout the rest of the curriculum. This is of importance because cannabis is often used by patients to treat symptoms of illness other than psychological symptoms. Universities should be aware of this dilemma and perhaps consider conducting an internal survey with students to estimate the current amount of cannabis education and identify areas or disciplines that students’ feel should be more comprehensive in their teaching. Additionally, perusing the curricula of other medical schools both locally and abroad may provide more insight into the cannabis education of doctors globally.

A factor that may contribute to this difficulty is the relatively sparse database that exists for the medicinal use of cannabis in most conditions where its use is regularly advocated for. A few cannabinoids (dronabinol, nabilone) with high-quality evidence have been approved by the US Food and Drug Administration (FDA) for chemotherapy-induced nausea and vomiting as well as appetite stimulation in conditions that cause weight loss, and more recently, cannabidiol was approved for the treatment of Dravet syndrome and Lennox-Gastaut syndrome. Chronic pain, muscle spasticity, tic disorders and Parkinson disease are major areas of research in the medical cannabis sphere with varying current evidence.^17^

This study also investigated the possible influence that prior experience with cannabis could have on the views and/or opinions expressed by a participant. The study found that prior cannabis use did have a relationship with more permissive and positive views about the substance and that the more frequent participants had used cannabis the more favourable their views tended to be. These findings are consistent with results from international studies showing that individuals with prior substance use, including cannabis, tend to perceive a lower risk of adverse effects related to the substance.^18^

The main limitation of this study would be the small sample group used. The study was originally designed to be conducted using the entire group of SIC students, but in accordance with COVID-19 precautions, the sampling strategy had to be altered. It has been shown that medical students are a relatively homogenous group,^19^ but a larger sample size could have provided a more varied response to questionnaires. Further limitations can be drawn when assessing the internal consistency of the questionnaire and the fact that it was validated using face validity. Another limitation was the use of the rather dichotomous response of agree or disagree on the questionnaire, when a Likert scale with a scoring of five options would have had more utility. Lastly, when assessing the sources from where students obtained information about cannabis, opportunity should have been provided for multiple sources of information. This is key because in reality people will most commonly use more than one source and the sources themselves could then have been grouped into primary and secondary sources.

The second limitation could be the possible underreporting of cannabis use by students because of the stigma attached to cannabis use. However, it is important to note that this study used the principles of voluntary participation and complete anonymity to curtail this concern.

Future studies should be conducted once the cannabis laws in South Africa have been finalised and larger samples of participants can be used. However, this particular study captured a unique moment in time because of its topical nature, taking into account how recently South African cannabis legislature has evolved.

## Conclusion

In summary, the study echoes certain similar points made by international studies. Cannabis laws are getting less restrictive globally. This means a possible increase in cannabis use in all patient populations and accordingly medical professionals will have to be ready to guide patients and offer advice regarding cannabis. More emphasis should be placed on the subject during the training of medical professionals, both in a clinical setting and classroom setting, seeing as current teaching practices have left the majority of students feeling unprepared to assist or advise patients.
